# Actionable Pharmacogenomics and Essential Medicines: An Analysis of WHO and African Lists for Safer and Efficacious Drug Use

**DOI:** 10.1002/cpt.70268

**Published:** 2026-04-03

**Authors:** Tinashe A. Mazhindu, Mohamed Nagy, David Twesigomwe, Gaye Agesa, Janine Scholefield, Collen Masimirembwa

**Affiliations:** ^1^ African Institute of Biomedical Science and Technology Harare Zimbabwe; ^2^ Department of Oncology, Medical Physics and Imaging Sciences, Faculty of Medicine & Health Sciences University of Zimbabwe Harare Zimbabwe; ^3^ Department of Pharmaceutical Services and Sciences Children's Cancer Hospital Cairo Egypt; ^4^ Personalized Medication Management Unit Children's Cancer Hospital Cairo Egypt; ^5^ Sydney Brenner Institute for Molecular Bioscience, Faculty of Health Sciences University of the Witwatersrand Johannesburg South Africa; ^6^ African Population and Health Research Center Nairobi Kenya; ^7^ Bioengineering and Integrated Genomics Group Council for Scientific and Industrial Research Pretoria South Africa; ^8^ Division of Human Genetics National Health Laboratory Service, and School of Pathology, Faculty of Health Sciences, University of the Witwatersrand Johannesburg South Africa

## Abstract

The World Health Organization Model Essential Medicines List and African national essential medicines lists (EMLs) detail drugs intended to be consistently available within national health systems, thereby supporting clinicians and pharmacists in making evidence‐based treatment decisions. At present, these EMLs do not account for pharmacogenomics, despite known drug‐gene interactions and the considerable genetic diversity found throughout Africa. In the absence of pharmacogenomic considerations, the medicines lead to sub‐optimal treatment outcomes, especially across a continent such as Africa which represents more genetic variation that the rest of the world combined. Herein, we review EMLs from the WHO, and from 52 African countries, highlighting systemic medicines for which actionable pharmacogenomic‐biomarker testing recommendations exist. Furthermore, we assess the feasibility of PGx‐guided dosing in eight African countries by examining the availability of registered drug formulations that facilitate dose adjustment. The 2023 WHO EML comprised 447 unique systemic medicines, with 58 (13%) categorized as medicines with actionable pharmacogenomic biomarkers. African country EMLs collectively featured 774 such medicines, with an average of 294 per country (range: 125–465). On average, for each country one in every eight medicines in the Essential Medicines List was associated with established pharmacogenomic guidance. PGx recommendation. Cumulatively in African EMLS the most frequent drug classes with this designation are anti‐infectives (25.6%), immunomodulators/antineoplastics (16.7%), and medicines for mental and behavioral disorders (14.1%). Analysis of eight African countries determined that implementability ranged from 75% to 96% based on product formulation and strength availability to enable drug or dose modification. We provide data that contributes towards a foundation of increasing evidence showing that integrating pharmacogenomic knowledge into the selection processes for essential medicines and ensuring the availability of appropriate drug formulations can enhance treatment safety and efficacy across Africa.

**Study Highlights**

**WHAT IS THE CURRENT KNOWLEDGE ON THE TOPIC?**

Current evidence indicates that the use of pharmacogenomic biomarker‐guided therapies enhances patient outcomes with respect to both safety and efficacy. Additionally, African has the widest genetic diversity in the world.

**WHAT QUESTION DID THIS STUDY ADDRESS?**

Identify medicines on the WHO Model Essential Medicines List and African national essential medicines lists that have actionable pharmacogenomic‐biomarker testing recommendations, specify their WHO‐assigned drug classes and associated pharmacogenes, and assess the feasibility of implementing dose modifications based on the characteristics of registered medicines.

**WHAT DOES THIS STUDY ADD TO OUR KNOWLEDGE?**

Understanding each country's essential medicines list within the framework of pharmacogenomics may enhance the safety and efficacy of medical treatments, while also identifying clinical implementation challenges.

**HOW MIGHT THIS CHANGE CLINICAL PHARMACOLOGY OR TRANSLATIONAL SCIENCE?**

The study results inform the formulation pharmacogenomic implementation strategy in different African countries and identify research gaps needing attention.

## BACKGROUND

Essential medicines are vital drugs that meet the priority healthcare needs of a given population safely and effectively.^1^ The first World Health Organization (WHO) model Essential Medicines List (EML) was published in 1977. This list has been reviewed every 2 years since and provides a template for adaptation by national governments into their own National Essential Medicine Lists (NEMLs).^2^ Approximately 150 countries worldwide have NEMLs compiled based on the WHO model EML.^3^ NEMLs outline the medicines that should be available in a given national health system at all times for all people, guiding clinicians and pharmacists in evidence‐based rational drug usage.^1^ The primary principles guiding drug selection are public health relevance, evidence of benefits and harms, and consideration of costs and affordability.^4^ There are substantial differences in the medicines selected for NEML globally, and across Africa, due to social, demographic, economic, political, geographical, developmental, and epidemiological factors.^5^, ^6^, ^7^ The medicine selection process documented by the WHO has not specifically considered pharmacogenomics (PGx) and its role in safety and efficacy challenges. PGx research focuses on determining the impact of genetic variations on the pharmacokinetics and/or pharmacodynamics of medicines and the consequential altered risk of toxicity or diminished efficacy.^8^ Given that evidence for drug safety, harms, and benefits in a population must be continuously surveyed and improved with all the latest data, PGx is a potential key pillar additionally in the collation of the EMLs.^9^, ^10^

African nations are at different stages of development and are currently undergoing significant changes in disease burden distribution, while also possessing the greatest genetic diversity globally.^11^, ^12^, ^13^ Most of these factors have a bearing on the selection and prescribing requirements in African NEMLs. At present, compared to the Global North, very limited drug development is taking place in Africa and far fewer clinical trials include continental African participants. Considering these disparities, as well as the high genetic diversity in Africa compared to non‐African biogeographical groups, African nations face a significant challenge in drug safety and efficacy. Examples of this are well documented for drugs such as efavirenz (neuropsychiatric side effects). However, PGx information such as this has not been incorporated into listing descriptions at present, despite recent annotations and drug dosing recommendations by the various research consortiums like Clinical Pharmacogenetics Implementation Consortium (CPIC) in the USA, Dutch Pharmacogenetics Working Group (DPWG) in Europe, and other consortia.^2^ While PGx focuses on individual benefits for personalized medicine, considering genotype and phenotype frequencies in determining African NEMLs could enhance safety and efficacy of chosen interventions at a national level before focusing on individuals.^14^ This benefit would apply to both drug selection and the range of drug formulations available within a country, enabling clinical teams to adjust dosages as needed. In the UPGx‐Prepare study conducted in Europe, pharmacogenomics implementation reported a 30% reduction in the odds of adverse drug reactions.^15^ Pre‐emptive pharmacogenomic‐biomarker testing is a cost‐effective strategy with potential for greater impact in resource‐limited countries.^16^

Identifying medicines with actionable pharmacogenomic‐biomarker (MAPB) testing recommendations in the WHO EML and NEMLs can guide African researchers and provide valuable information to health policymakers, particularly those on NEML selection committees.^8^, ^17^, ^18^ We conducted this study to identify the MAPB in the WHO EML and African NEMLs, compare these across African regions, analyze relevant drug classes and associated pharmacogenes for continent‐wide focus and determine implementability of pharmacogenomic‐biomarker guided interventions based on availability of registered drug formulations.

## MATERIALS AND METHODS

We conducted a descriptive study by searching the WHO repository of NEMLs (https://www.who.int/teams/health‐product‐policy‐and‐standards/assistive‐and‐medical‐technology/essential‐medicines/national‐emls) which is a regularly updated portal for NEMLs, national formularies, and national standard treatment guidelines as of 1 June 2025. The repository contained 144 unique country documents at the time of download. The latest WHO EML and each country's most recent African NEMLs were downloaded from the repository. We excluded incomplete NEML documents i.e. a standard treatment guideline without essential medicines lists, and those not downloadable from the repository.

### Data collection processes

A data extraction tool was created: one investigator extracted data from the WHO EML and each African country, and another investigator verified it before database creation for analysis. Collected data included country name, African region, exact document title, publication year, edition, counts of systemic medicines and MAPBs (from CPIC, DPWG, or other guidelines), drug classification per WHO EML, and corresponding pharmacogenes for testing. Medicines were grouped by their English non‐proprietary generic drug names, with all salt formulations recorded as a single entity (e.g., diclofenac potassium and diclofenac sodium were recorded as diclofenac).

The study analyzed MAPB among systemic non‐vaccine medicines only, excluding products not administered for systemic effect. Medicines and medical products on the lists were excluded if they were classified under therapeutic foods, blood products, plasma substitutes, dermatological agents, antiseptics, disinfectants, immunologicals, vaccines, ophthalmic preparations, dialysis solutions, electrolyte and acid–base correction solutions, vitamins, minerals, local ENT and dental preparations, reproductive health devices, or oral rehydration formulas. MAPB determination was conducted using a curated list of MAPBs and their corresponding pharmacogenes, based on CPIC, DPWG, and other relevant guidelines as of 1 January 2025 using the ClinPGx clinical guideline annotations (https://www.clinpgx.org/guidelineAnnotations). The MAPBs list curated for this study comprised only those medicines with an actionable pharmacogenomic biomarker and a corresponding testing recommendation in any of the guidelines. Medicines annotated without a testing recommendation were excluded from consideration. For the WHO EML and each NEML, the presence or absence of MAPB was determined systematically.

We assessed the implementability of pharmacogenomic‐biomarker guided interventions, based on the availability of registered drug formulations, in eight African countries with a National Regulatory Authority (NRA) recognized at WHO Global Benchmarking Tool Maturity Level 3 (ML3): Egypt, Ghana, Nigeria, Rwanda, South Africa, Senegal, Tanzania, and Zimbabwe.^19^ For each MAPB in every NEML, we downloaded all registered medicine brands along with their formulations and strengths from each country's list of registered human medicines. **Table** **S1** below provides a list of eight NRAs, along with the corresponding websites and access links used for this study as of 15/01/2025. Implementability was considered feasible if a registered product's formulation and strength allowed dose adjustments according to PGx guidelines. For instance, if the normal dose of drug X is 100 mg daily but a certain phenotype requires 50 mg and only a 100 mg capsule is available, the recommendation is unimplementable. If, however, a 50 mg formulation or an oral suspension is registered in the country, the recommendation is implementable. This process was applied to each MAPB in each of the eight ML3 countries. All medicines with a recommendation to use an alternative were assumed to be feasible along with all medicines for intravenous administration in dose modification in this analysis.

### Data analysis

For descriptive data, we calculated means, ranges, and proportions as percentages and ratios. Comparison of EML against the MAPBS list from the guidelines was performed systematically using excel functions. The Shapiro–Wilk test was used to determine if data were normally distributed, and ANOVA statistical test was used to compare the means between African region means. A *P*‐value < 0.05 was considered significant.

## RESULTS

### Description of essential medicines lists

The WHO repository contained 144 NEMLs, including 54 from African countries, with 52 meeting the inclusion criteria. The NEML documents for Algeria and Malawi were incomplete, resulting in their exclusion from the analysis. Fourteen African NEML (27%) were published after the 23rd WHO EML of 2023, with the average publication year being 2019 (range: 2005–2024). **Table** **1** shows a summary description of the NEML reviewed, the total number of systemic medicines and MAPBs listed, and the NRA ML3 status of each country.

**Table 1:** Characteristics of World Health Organization Model List and African National Essential Medicines Lists reviewed in the study.

Country	Region	Name of Essential Medicines List publication (exact name)	Year (Edition)	Total number of systemic medicines listed	Total number of MAPB listed per NEML/WHO EML (% of Total)	National Regulatory Authority ML3 Status (No/Yes)
WHO	Global	Model List of Essential Medicines	2023 (23^rd^)	447	58 (13)	No
Angola	Southern Africa	Lista Nacional de Medicamentos Essenciais	2021	248	33 (13)	No
Benin	Western Africa	Liste Nationale Des Medicaments Essentiels Enfants et Adultes	2018 (8^th^)	372	45 (12)	No
Botswana	Southern Africa	Botswana Essential Medicines List	2016 (3^rd^)	302	41 (14)	No
Burkina Faso	Western Africa	Liste Nationale Des Medicaments Essentiels Et Autres Produits De Sante	2023	353	44 (12)	No
Burundi	Central Africa	Liste Nationale Des Medicaments Essentiels Au Burundi	2022	252	30 (12)	No
Cabo Verde	Western Africa	Lista Nacional de Medicamentos Essenciais	2018	266	36 (14)	No
Cameroon	Central Africa	Liste Nationale De Medicaments Et Autres Produits Pharmaceutiques Essentiels	2022	366	39 (11)	No
Central Africa Republic	Central Africa	Liste Nationale Des Medicaments Essentiels Et Dispositifs Medicaux	2017	273	35 (13)	No
Chad	Central Africa	Liste Nationale Des Medicaments Essentiels Et Autres Produits De Sante	2022	368	45 (12)	No
Comoros	Eastern Africa	Liste Nationale Des Medicaments Essentiels	2020	252	25 (10)	No
Côte d'Ivoire	Western Africa	Liste Nationale De Medicaments Et Du Materiel Biomedical Pharmaceutiques Essentiels	2024	413	43 (10)	No
Djibouti	Eastern Africa	Liste Djiboutienne Des Medicaments Essentiels	2016	180	23 (13)	No
DRC	Central Africa	Liste Nationale des Médicaments Essentiels	2020	276	33 (12)	No
Egypt	North Africa	Egyptian Essential Drug List	2019	350	42 (12)	Yes
Equatorial Guinea	Central Africa	Lista Nacional De Medicamentos Esenciales De Guinea Ecuatorial	2012	125	16 (13)	No
Eritrea	Eastern Africa	Eritrean National List of Medicines	2010 (5^th^)	235	29 (12)	No
Eswatini	Southern Africa	Essential Medicine List	2012	236	35 (15)	No
Ethiopia	Eastern Africa	Ethiopian Essential Medicines List	2024	410	49 (12)	No
Gabon	Central Africa	Liste Nationale Des Medicaments Et Dispositifs Medicaux Essentiels	2024	249	29 (12)	No
Gambia	Western Africa	Gambia Essential Medicine List	2024	182	28 (15)	No
Ghana	Western Africa	Essential Medicines List	2017 (7^th^)	306	35 (12)	Yes
Guinea	Western Africa	Liste Nationale Des Medicaments Essentiels	2021 (7^th^)	251	29 (12)	No
Guinea‐Bissau	Western Africa	Lista De Medicamentos Esenciales	2024	322	40 (12)	No
Kenya	Eastern Africa	Kenya Essential Medicines List	2023	444	49 (11)	No
Lesotho	Southern Africa	Lesotho Essential Medicines List	2005	141	21 (15)	No
Liberia	Western Africa	Liberia Essential Medicines List	2023	274	40 (15)	No
Libya	North Africa	Libyan Essential Medincies List	2019 (1^st^)	433	48 (11)	No
Madagascar	Eastern Africa	Liste Nationale des Médicaments Essentiels et Intrants de Santé	2019 (6^th^)	327	42 (13)	No
Mali	Western Africa	Liste Djiboutienne Des Medicaments Essentiels	2024	199	24 (12)	No
Mauritania	North Africa	Liste Nationale des Médicaments Essentiels	2024	363	45 (12)	No
Mauritius	Eastern Africa	Approved Drug List For Public Hospitals In Mauritius	2022	300	41 (14)	No
Morocco	North Africa	Nomenclature Nationale De Medicaments Essentiels	2020	296	42 (14)	No
Mozambique	Southern Africa	Lista Nacional De Medicamentos Esenciales	2017	223	34 (15)	No
Namibia	Southern Africa	Namibia Essential Medicines List	2021 (7^th^)	217	26 (12)	No
Niger	Western Africa	Liste Nationale Des Medicaments Essentiels	2018	184	22 (12)	No
Nigeria	Western Africa	Nigeria Essential Medicines List	2020 (7^th^)	335	44 (13)	Yes
Republic of Congo	Central Africa	Liste Nationale Des Medicaments Essentiels	2016 (7^th^)	263	32 (12)	No
Rwanda	Eastern Africa	National List Of Essential Medicines For Adults	2022 (14^th^)	321	43 (13)	Yes
Sao Tome and Principe	Central Africa	Lista Nacional de Medicamentos	2020	432	48 (11)	No
Senegal	Western Africa	Liste Nationale De Medicaments Et Produits Essentiels Du Senegal	2022	328	36 (11)	Yes
Seychelles	Eastern Africa	List of Essential medicines	2024	247	34 (14)	No
Sierra Leone	Western Africa	National Essential Medicines List	2021	243	32 (13)	No
Somalia	Eastern Africa	Somali Essential Medicines List	2019	244	40 (16)	No
South Africa	Southern Africa	Essential Medicines Lists for South Africa	2023	346	46 (13)	Yes
South Sudan	Eastern Africa	South Sudan Essential Medicines List	2018	275	44 (16)	No
Sudan	Eastern Africa	Sudan National Essential Medicines List	2014	411	54 (13)	No
Tanzania	Eastern Africa	National Essential Medicines List	2021	358	46 (13)	Yes
Togo	Western Africa	Liste Nationale Des Médicaments Essentiels	2021	174	26 (15)	No
Tunisia	North Africa	Formulaire Therapeutique Tunisien	2012 (3^rd^)	465	50 (11)	No
Uganda	Central Africa	Essential Medicines and Health Supplies List for Uganda	2023	339	46 (14)	No
Zambia	Southern Africa	Zambia Essential Medicines List	2020	258	37 (14)	No
Zimbabwe	Southern Africa	Essential Drug List Of Zimbabwe	2020 (8^th^)	301	44 (15)	Yes

**Figure 1:**
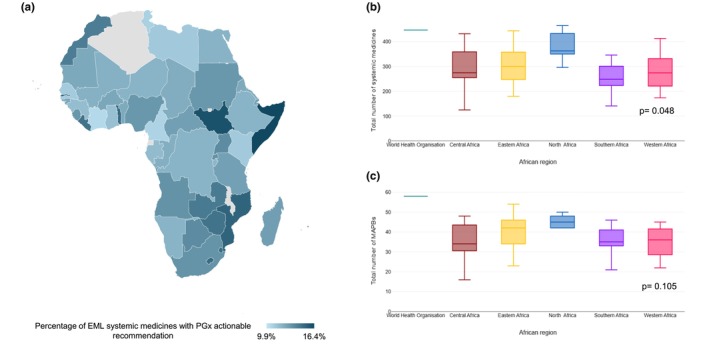
Essential medicines from the WHO 23^rd^ Model List and African National Lists. (**a**) Proportion of essential medicines that have a pharmacogenomic testing recommendation, per country. No data for Malawi and Algeria. (**b**) Total number of systemic medicines listed, compared regionally. (**c**) Total number of medicines with actionable pharmacogenomic biomarkers (MAPBs) listed compared regionally.

The WHO EML includes 447 unique systemic medicines, whereas NEMLs collectively list a total of 774 unique systemic medicines. On average, the NEMLs for African countries contain 294 unique systemic medicines, ranging from 125 to 465. Equatorial Guinea had the lowest number of systemic medicines, while Tunisia had the highest. A statistically significant difference is observed between African regions (*P* = 0.048) with North African countries having more systemic medicines in their respective NEML compared to other regions, and Southern Africa having the least, as shown in **Figure** **1** **b**.

### Medicines with actionable pharmacogenomic‐biomarkers

There were 447 systemic medicines in the WHO EML, and 58 (13%) of these are MAPBs. Among African NEMLs the total number of MAPBs per country ranged from 16 to 54 (average 38). Equatorial Guinea has the lowest number of MAPBs, while Libya, São Tomé and Príncipe, Kenya, Ethiopia, and Tunisia together have the most MAPBs, as shown in **Table** **2**. MAPBs constitute 9.9%–16% of systemic medicines across the continent's NEMLs, with Comoros having the lowest proportion at 10%, and the countries of Togo, Mozambique, Gambia, South Sudan, and Somalia all have the highest of 16%, as illustrated in **Figure** **1** **a**.

**Table 2:** Medicines with actionable pharmacogenomic‐biomarker listed in African National Essential Medicines Lists.

Medicine	Totals of countries listing the medicines	Percentage (%)
Carbamazepine	52	100
Ibuprofen	52	100
Abacavir	51	98
Gentamicin	51	98
Omeprazole	51	98
Efavirenz	50	96
Amitriptyline	50	96
Haloperidol	50	96
Allopurinol	49	94
Dapsone	47	90
Fluorouracil	45	87
Phenytoin	45	87
Doxorubicin	44	85
Tamoxifen	44	85
Amikacin	43	83
Atazanavir	43	83
Tramadol	43	83
Isoflurane	42	81
Ondansetron	42	81
Codeine	42	81
Cisplatin	41	79
Azathioprine	41	79
Halothane	39	75
Suxaméthonium	38	73
Warfarin	38	73
Clopidogrel	37	71
Nitrofurantoin	36	69
Risperidone	36	69
Clomipramine	34	65
Atorvastatin	33	64
Mercaptopurine	33	64
Daunorubicin	31	60
Streptomycin	30	58
Simvastatin	30	58
Capecitabine	30	58
Lamotrigine	30	58
Sevoflurane	29	56
Irinotecan	25	48
Primaquine	24	46
Flucytosine	23	44
Kanamycin	23	44
Flucloxacillin	22	42
Imipramine	21	40
Tacrolimus	19	37
Thioguanine	19	37
Methylene blue	17	33
Metoprolol	16	31
Sertraline	15	29
Voriconazole	14	27
Citalopram	14	27
Paromomycin	13	25
Peginteferon alfa‐2a	13	25
Rosuvastatin	13	25
Acénocoumarol	12	23
Escitalopram	12	23
Paroxetine	12	23
Quetiapine	12	23
Zuclopenthixol	11	21
Lansoprazole	9	17
Pantoprazole	9	17
Aripiprazole	9	17
Celecoxib	6	12
Piroxicam	5	10
Peginteferon alfa‐2b	4	8
Fluodione	4	8
Meloxicam	4	8
Fluvastatin	3	6
Pravastatin	3	6
Rasburicase	3	6
Pimozide	3	6
Neomycin	2	4
Atomoxetine	2	4
Fosphenytoin	1	2
Lornoxicam	1	2

A comparison across African regions showed no statistically significant difference between the different groups Central Africa, Eastern Africa, North Africa, Southern Africa, and Western Africa in terms of total number of MAPBs (*P* = 0.105) (**Figure** **1** **c**).

**Figure 2:**
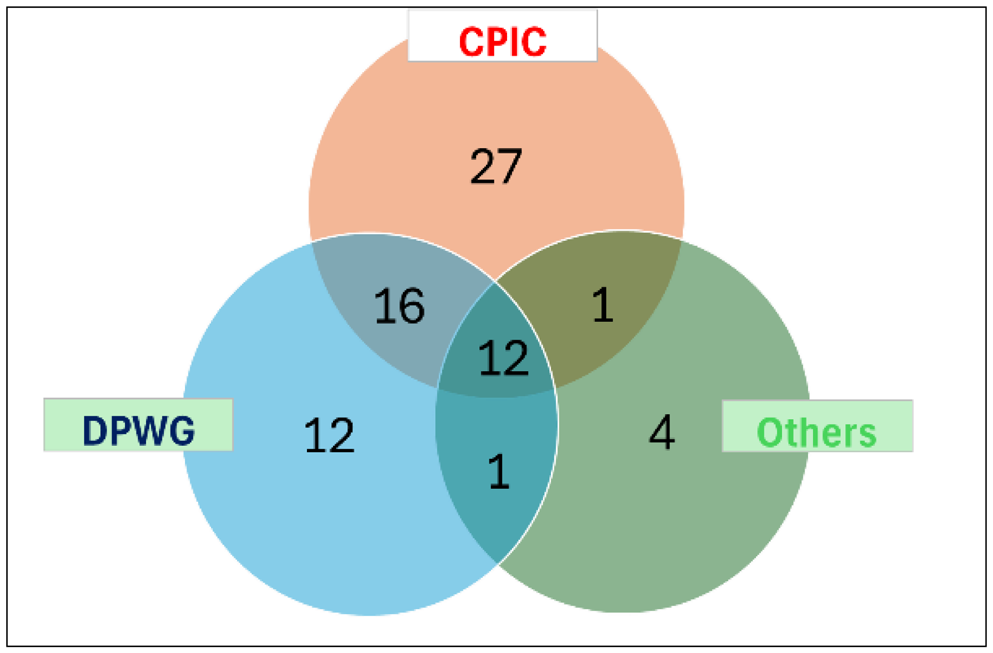
Venn diagram illustrating the distribution of 74 medicines with actionable pharmacogenomic biomarkers listed in the African National Essential Medicines List, categorized by source of recommendation. CPIC, Clinical Pharmacogenetics Implementation Consortium; DPWG, Dutch Pharmacogenetics Working Group.

**Figure 3:**
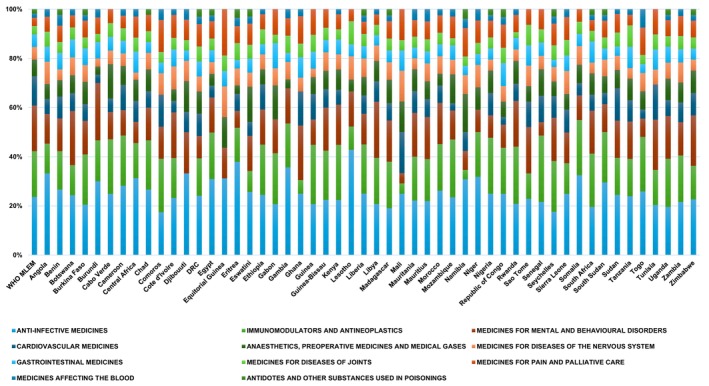
A graph illustrating the drug classes of medicines that include actionable pharmacogenomic biomarker testing recommendations across various African National Essential Medicines Lists and the World Health Organization Model List.

A total of 74 MAPBs are listed in at least one African NEML (**Table** **2**). All 74 MAPBs along with the proportion of African NEMLs that list each entity and the intersections of the consortium guidelines reveal that the CPIC consortium, DPWG group, and other consortia account for 56 (76%), 41 (55%), and 18 (24%) of the recommendations, respectively (**Figure** **2**). The 74 MAPBs in African NEMLs span 11 drug classes as classified by the WHO. The most common drug classes incorporating MAPBs for the African NEMLs are anti‐infective medicines (25.6%), immunomodulators and antineoplastics (16.7%), mental and behavioral disorder medicines (14.1%), pain and palliative care medicines (7.8%), anesthetics and preoperative medicines (7.8%), and nervous system disease medicines (6.7%) (**Figure** **3**).

**Figure 4:**
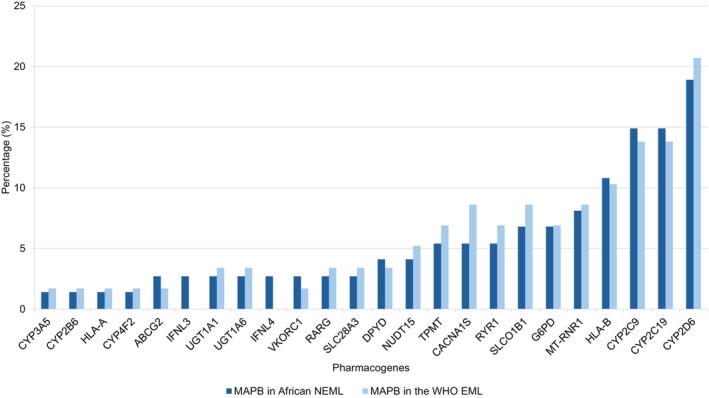
Distribution and comparison of the corresponding pharmacogenes for the medicines with actionable pharmacogenomic‐biomarkers in the WHO MLEM and the combined African NEMLs.

There are 22 corresponding pharmacogenes for the MAPBs in the WHO EML while there are 24 in the collective African NEMLs. MAPBs on the latest WHO EML no longer include peginterferon alfa‐2a and alfa‐2b, which are MAPBs corresponding to the pharmacogenes IFNL3 and 4. However, as shown in **Figure** **4**, peginterferon alfa‐2a and peginterferon alfa‐2b are listed in 23% and 7.7% of African NEMLs, respectively. A summary of the WHO EML and African NEML corresponding pharmacogenes for the MAPBs is shown in **Figure** **4**.

To evaluate implementability based on the criteria described above, the human medicines register lists of eight African countries were assessed. An analysis of implementability, considering the availability of registered brands, formulations, and product strengths in eight ML3‐rated African countries, indicated that 75–96% of these countries had products available to support PGx‐biomarker guided dose adjustments. Egypt and South Africa showed the highest implementability, with 96% of MAPB on the NEML having suitable formulations for PGx implementation, while Nigeria had the lowest rate at 75% (**Figure** **S1**). The dataset generated for this analysis comprising the medicines names, nature of recommendation (i.e. use alternative drug, dose modification or both), total registers brands, formulations available and the lowest and highest product strengths available, and our conclusion on implementability of the recommendation and the reason for the assigned conclusion is accessible at https://doi.org/10.17632/zyd7vcnkyr.1.

## DISCUSSION

Most African NEMLs have over 200 systemic medicines listed, and this is consistent with the global trend.^7^ In our study, we found that a significant number of the drugs in the WHO EML and African NEMLs were MAPBs spanning up to 11 drug classes corresponding to 24 pharmacogenes.^7^ On average, approximately one in eight (12.9%) drugs listed in African NEMLs have a pre‐emptive pharmacogenomic‐biomarker testing recommendation meaning a significant number of patients may be receiving medical interventions where safety and toxicity outcomes may be compromised but an opportunity exists to improve this in a cost effective manner in resource limited countries.^15^, ^16^ Additionally, in the eight African countries with ML3 NRAs where registered drug products were assessed, the feasibility of implementing PGx‐guided dose adjustments was generally achievable, although it differed among countries and improvements are needed.

The fundamental aim of an NEML is to streamline medicine procurement and distribution, lowering costs for public health systems and patients.^18^ The stated objectives of NEMLs are to promote rational drug use and support better health outcomes. Compiling and implementing an NEML further achieves broader goals, whilst helping public health systems by guiding medicine procurement and distribution, and in some countries it forms a pillar in planning health insurance, directing charitable medicine donations, and prioritizing local medicine production.^20^ Several studies have highlighted challenges with NEMLs that include inconsistencies with availability, exorbitantly high costs of new essential medicines, especially in oncology and non‐communicable diseases, and the continued risk of developing antimicrobial resistance due to inappropriate use of anti‐infective medicines.^2^, ^21^ Studies on challenges with selecting and implementing NEMLs in African countries have tended to focus on access and affordability, and medicine safety is seen mainly through the lens of authentic drug procurement, shipment, and storage.^22^, ^23^, ^24^ NEMLs vary widely in size across the continent as observed in this study, with North Africa having the largest lists by numbers. Despite a generally similar disease trend and profiles in Africa, local priorities, development partner influence, economic resources, health expenditure, NEML purpose and assigned importance, health infrastructure, medicines regulations, and geographic and ethnic factors contribute to these differences. Notably, non‐communicable and infectious disease rankings differ between North Africa and Sub‐Saharan Africa.^5^, ^6^, ^7^

In this study we show that medicine safety and efficacy in an NEML especially in Africa can benefit from incorporating pharmacogenomic into the consideration matrix and in accompanying assays.^25^ In our previous review article we showed that in the African population, MAPB‐recommended changes in medicine selection of dose alteration for some medicines could affect up to 75% of the population.^8^ Coupling this African genotype/phenotype fact with the extensive presence of the MAPBs in African NEMLs reported in this study illustrates that a need for incorporating PGx exists and would be effectual in limiting sub‐optimal treatment outcomes.^26^, ^27^, ^28^ Bringing these benefits of the human genome knowledge to the continent can have an impact of efficient use of health and financial resources while expanding the field of African PGx research.^25^, ^29^, ^30^ In this study, we show that the reduction in adverse event risk that may emerge from implementing pre‐emptive pharmacogenomic‐biomarker guided interventions could find fertile ground in Africa based on the medicines in common use.^31^

The MAPBs observed in African NEMLs in this study are indicated in several diseases and conditions. The broad drug classes they belong to show that pre‐emptive testing will improve health outcome associated with medical treatments widely.^32^ This presents an opportunity in improved care in anesthesia, antidotes and other substances used in poisonings, treatment of infections, cardiovascular care, gastrointestinal symptom management, immunomodulation and cancer care, management of clotting disorders, pain and anti‐inflammatories and the treatment of nervous systems and mental and behavioral disorders. As Africa undergoes a disease burden transition period from predominantly communicable diseases to non‐communicable diseases, a pharmacogenomic integrating approach would be applicable to both groups of diseases.^13^, ^33^ Communicable diseases where MAPBs are indicated for use include acute and chronic infection, pediatric and adult infection, and viral, bacterial and parasitic infections, covering a litany of drug classes. Optimizing treatment related outcomes will naturally benefit a sizeable number of patients directly given the centrality of these medicines in HIV, malaria and tuberculosis treatment. Non‐communicable diseases like cardiovascular, psychiatric, and oncology conditions have key medicines included like statins, metoprolol, clopidogrel, sertraline, amitriptyline, haloperidol, tacrolimus, irinotecan, capecitabine, and tamoxifen among others.^34^, ^35^, ^36^, ^37^, ^38^ We found that the use of MAPBs across Africa varied but were not different in a statistically significant way by region. This allows this African MAPB list we have found to be considered jointly and effectively for incorporation in ongoing African regional harmonization of essential medicines and joint procurement like the Southern Africa Development Community and cancer treatment guidelines for Sub‐Saharan Africa.^39^, ^40^, ^41^

The implementation of PGx‐guided medical treatments requires the availability of alternative medications or appropriate dose adjustments, both of which can present significant challenges in some countries. This challenge has not received sufficient attention in PGx implementation research; however, it represents a limitation that could be addressed with increased focus.^42^ This study demonstrates that the range of drug formulations and brands registered within a country can serve as a limiting factor for effective implementation of dose modification as recommended in response to patient genotype/phenotypes. Successfully translating PGx knowledge from the laboratory to clinical practice hinges on the final step: dispensing the correct drug dosage incorporating their own genetics, a process highly dependent on the availability of suitable formulations and enabling product strengths. This limitation is mainly present on dose modifications rather than alternative drug use recommendations. African drug regulatory authorities can play a crucial role by reviewing the MAPBs registered in their jurisdictions and enacting policies that support the availability of drug formulations facilitating dose adjustments. The establishment of the African Medicines Agency presents an opportunity for this level of work to be undertaken at a continental level.^43^, ^44^, ^45^ Such measures would enhance both the safety and efficacy of patient treatments in a manner that prioritizes regional disease burdens.^43^

This is the first study to identify MAPBs across Africa, providing a foundation for future PGx research and application to widely used medicines on the continent. This study was limited by relying solely on the WHO repository to extract each country's systemic medicines list, facing challenges such as translation issues, inconsistent use of standard medicinal names (including some brand names), and the risk that newer NEML versions may not yet have been uploaded. A medicine's inclusion in an NEML does not guarantee its use; further research into drug purchases, adverse events, and national registrations would provide more insight. In assessing implementability, additional research focusing on specific drug classes and evaluating the availability of alternative drugs for indications will be necessary. Nonetheless, we believe the methodology highlights crucial areas for improving medical care in Africa through incorporating PGx.

## FUNDING

The study was funded by the Bill and Melinda Gates Foundation (grant INV‐058365) for the development of the target policy profile framework for integrating Africa's genomic heterogeneity in drug discovery, development, and deployment.

## CONFLICT OF INTEREST

The authors declared no competing interests for this work.

## AUTHOR CONTRIBUTIONS

TAM, MN, DT, GA, JS, CM wrote the manuscript; TAM, MN, DT, GA, JS, CM designed the research; TAM, MN, DT, GA, JS, CM performed the research; TAM, MN, DT, GA, JS, CM analyzed the data.

## ETHICAL CONSIDERATION

No specific direct individual or public involvement was conducted for this research.

## Supporting information


Figure S1.

Table S1.

## Data Availability

The entire data used in this study is publicly available, maintained and regularly updated for access by all. (2) The dataset generated for the implementability analysis for dose adjustments has been reposited at https://doi.org/10.17632/zyd7vcnkyr.1.
