# Supplementary material for: Actionable Pharmacogenomics and Essential Medicines: An Analysis of WHO and African Lists for Safer and Efficacious Drug Use
Source: Clin Pharmacol Ther. 2026 Apr 3;119(5):1371–81. doi: 10.1002/cpt.70268 (PMC13083375; doi:10.1002/cpt.70268)
Supplement: Supplementary file 1 — Figure S1. [file CPT-119-1371-s001.pdf]

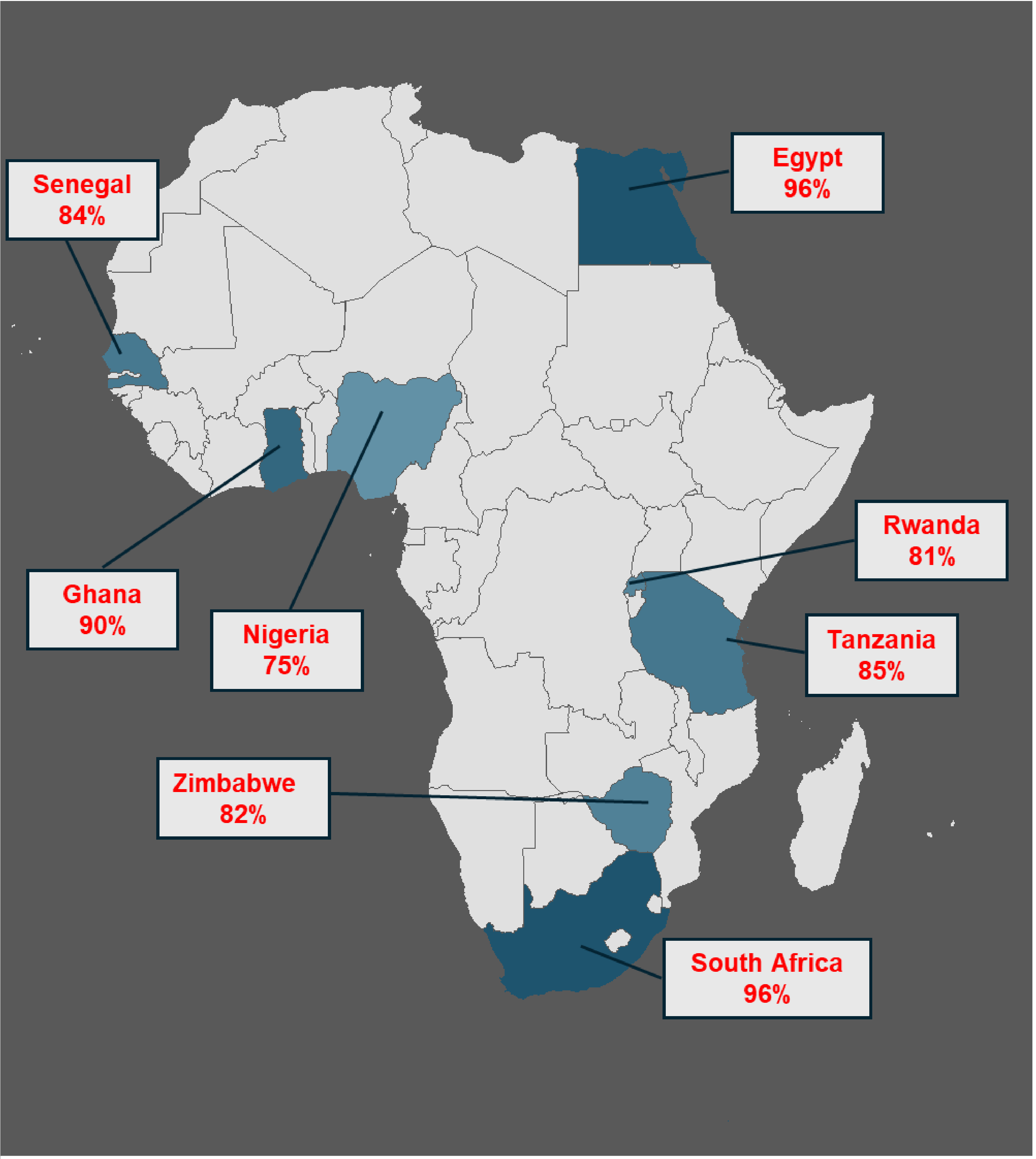

Figure S1: Map indicating the eight African countries whose National Regulatory Authorities are recognized at WHO Global Benchmarking Tool Maturity Level 3, and the percentage of medicines with actionable pharmacogenomic biomarkers listed in their respective national essential medicines lists that have approved brands, their product strength and formulations allowing for recommended dose adjustments
