# Supplementary material for: Actionable Pharmacogenomics and Essential Medicines: An Analysis of WHO and African Lists for Safer and Efficacious Drug Use
Source: Clin Pharmacol Ther. 2026 Apr 3;119(5):1371–81. doi: 10.1002/cpt.70268 (PMC13083375; doi:10.1002/cpt.70268)
Supplement: Supplementary file 2 — Table S1. [file CPT-119-1371-s002.docx]

**Supplementary Table S1: The eight African National Regulatory Authorities with Maturity Level 3**

| **Country** | **National Regulatory Authority name and**  **Website** | **Access Link to product register** |
| --- | --- | --- |
| **Egypt** | Egyptian Drug Authority  www. edaegypt.gov.eg | <http://eservices.edaegypt.gov.eg/EDASearch/SearchRegDrugs.aspx> |
| **Ghana** | Food and Drugs Authority (FDA)  www.fdaghana.gov.gh | <http://196.61.32.245:55/publicsearch> |
| **Nigeria** | National Agency for Food and Drug Administration and Control (NAFDAC)  www.nafdac.gov.ng | <https://nafdac.gov.ng/our-services/registered-products/> |
| **Rwanda** | Rwanda Food and Drug Authority (Rwanda FDA)  www.fdaghana.gov.gh | <https://rwandafda.gov.rw/human-medicinal-products-register/?by=All> |
| **South Africa** | South African Health Products Regulatory Authority (SAHPRA)  www.sahpra.org.za | <https://medapps.sahpra.org.za:6006/> |
| **Senegal** | Agence sénégalaise de Réglementation pharmaceutique (ARP)  www.arp.sn | <https://arp.sn/> |
| **Tanzania** | Tanzania Medicines and Medical Devices Authority (TMDA)  www.tmda.go.tz | <https://imis2.tmda.go.tz/#/public/registered-medicines> |
| **Zimbabwe** | Medicines Control Authority of Zimbabwe (MCAZ)  www.mcaz.co.zw | <https://onlineservices.mcaz.co.zw/onlineregister/frmAllophaticRegister.aspx> |

(registered drug lists downloaded as of 15/01/2025)
